# Call Transmission Efficiency in Native and Invasive Anurans: Competing Hypotheses of Divergence in Acoustic Signals

**DOI:** 10.1371/journal.pone.0077312

**Published:** 2013-10-14

**Authors:** Diego Llusia, Miguel Gómez, Mario Penna, Rafael Márquez

**Affiliations:** 1 Fonoteca Zoológica, Departamento de Biodiversidad y Biología Evolutiva, Museo Nacional de Ciencias Naturales (CSIC), Madrid, Spain; 2 Programa de Fisiología y Biofísica, Instituto de Ciencias Biomédicas, Facultad de Medicina, Universidad de Chile, Santiago, Chile; Clemson University, United States of America

## Abstract

Invasive species are a leading cause of the current biodiversity decline, and hence examining the major traits favouring invasion is a key and long-standing goal of invasion biology. Despite the prominent role of the advertisement calls in sexual selection and reproduction, very little attention has been paid to the features of acoustic communication of invasive species in nonindigenous habitats and their potential impacts on native species. Here we compare for the first time the transmission efficiency of the advertisement calls of native and invasive species, searching for competitive advantages for acoustic communication and reproduction of introduced taxa, and providing insights into competing hypotheses in evolutionary divergence of acoustic signals: acoustic adaptation vs. morphological constraints. Using sound propagation experiments, we measured the attenuation rates of pure tones (0.2–5 kHz) and playback calls (*Lithobates catesbeianus* and *Pelophylax perezi*) across four distances (1, 2, 4, and 8 m) and over two substrates (water and soil) in seven Iberian localities. All factors considered (signal type, distance, substrate, and locality) affected transmission efficiency of acoustic signals, which was maximized with lower frequency sounds, shorter distances, and over water surface. Despite being broadcast in nonindigenous habitats, the advertisement calls of invasive *L. catesbeianus* were propagated more efficiently than those of the native species, in both aquatic and terrestrial substrates, and in most of the study sites. This implies absence of optimal relationship between native environments and propagation of acoustic signals in anurans, in contrast to what predicted by the acoustic adaptation hypothesis, and it might render these vertebrates particularly vulnerable to intrusion of invasive species producing low frequency signals, such as *L. catesbeianus*. Our findings suggest that mechanisms optimizing sound transmission in native habitat can play a less significant role than other selective forces or biological constraints in evolutionary design of anuran acoustic signals.

## Introduction

Invasive species are drivers of ecological and evolutionary changes [1–6] and a leading cause of the current biodiversity decline in all biomes [7–11]. Examining major traits that drive a taxon to become a widespread nonindigenous species and that favour invasion has been a key and long-standing goal of invasion biology [12–20]. Among other common features, organisms establishing and spreading outside their native range tend to show a high dispersal and reproductive capacity in diverse habitats and ecosystems [17,21–24]. 

Acoustic communication performs a key function in sexual selection and reproduction in a diversity of animal taxa, including insects, anurans, birds, and mammals (e.g. [25–28]). In these groups, the range of effective communication among conspecifics is constrained by the attenuation and degradation of the acoustic signals in the environments [29–41]. Sound transmission in natural conditions imposes alterations in both call amplitude and fidelity that may compromise the integrity of the signal reaching the receiver. Thus, species that produce advertisement calls maximizing propagation distance to reach potential recipients would presumably broaden their communication range and increase the probability of reproduction. 

In spite of the growing interest in studying the harmful effects of biological invasions [2–6,8–11], little attention has been paid to the possible consequences of acoustic intrusion of nonindigenous species in native species and communities. Recently, two studies have provided the first evidence of acoustic niche displacement generated by invasive species. Both & Grant [42] reported the effect of calls of the American Bullfrog *Lithobates catesbeianus* causing an increase in call frequency in *Hypsiboas albomarginatus* and Farina et al. [43] examined potential masking interferences of Red-billed Leiothrix *Leiothrix lutea* in a temperate bird community. Here we compare for the first time the relative efficiency of transmission of the advertisement calls of native and invasive species in different environments, searching for competitive advantages for acoustic communication and reproduction of introduced taxa.

Because conveying information is the basic function of sender-receiver communication systems, the assessment of signal transmission efficiency is relevant for gaining insights into the relative significance of concomitant influences driving the evolution of animal acoustic signals. Processes of divergence of species-specific advertisement calls are still poorly understood [44], and it has been proposed that the comparative approach contributes to the understanding of evolutionary patterns, shedding light on the environmental sources of divergent selection [45,46]. Accordingly, examining alterations of acoustic signals as they propagate in native and nonindigenous environments enables us to test competing hypotheses for evolutionary divergence of acoustic signals: acoustic adaptation [31,35] vs. morphological constraints [47,48]. To the best of our knowledge, no previous studies have explored this comparative approach with native and invasive species.

As predicted by the acoustic adaptation hypothesis [31,35], selective pressures lead to a signal design that favours sound transmission in the native habitats of species communicating by means of sounds. Evidence of local adaptation of acoustic signals to physical settings of the natural environments have mainly been found in birds and mammals (e.g. [30,40,49–60]). According to this hypothesis, it is expected that the advertisement calls of nonindigenous species exhibit suboptimal propagation, implying an *a priori* reproductive disadvantage relative to native species. 

Nonetheless, the design of animal acoustic signals is subjected to influences other than environmental, such as morphological constraints or phylogenetic inertia [44,47,48,61–65]. Thereby, smaller-sized individuals are limited to producing comparatively higher-pitched sounds [27,48,61,66,67], although such signals generally experience more attenuation over distance in natural environments, regardless of habitat structure (e.g. [32,34]). Because of such determinants, alternatively to the acoustic adaptation hypothesis it can be hypothesized that larger-sized invasive species would probably advertise by emitting signals with lower frequency and more efficient propagation than those of native species, although the calls of invasive species have not evolved in association with the particular habitat structure of their new environment.

In this study, we test the hypothesis that the advertisement call of an invasive anuran, the American Bullfrog *Lithobates catesbeianus*, propagates more efficiently than that of an Iberian anuran, the Perez’s Frog *Pelophylax perezi*, in native habitats of the latter species, providing the invasive species an advantage for acoustic communication and mate attraction. Using sound propagation experiments, we measured the attenuation rates of pure tones (0.2–5 kHz) and playback calls across four distances (1, 2, 4, and 8 m) and over two substrates (water and soil) in seven localities within the Iberian distribution range of *P. perezi*, including two localities in which *L. catesbeianus* were introduced, but failed to establish, in the past from abandoned commercial farms. These measurements provide a framework for the study of the effective communication range of both native and invasive species, and allow us to discriminate between two competing hypotheses that may explain the optimization of sound signal transmission and evolution of acoustic signals: 1) acoustic adaptation hypothesis, i.e. signal adaptation to local habitat conferring advantages to native species, vs. 2) morphological constraints hypothesis, i.e. size constraints of sound production mechanisms, permissive for invasive species advantages. Finally, we discuss how our findings contribute to the understanding of and the prediction of the impact of invasive species with mating systems based on acoustic communication.

## Materials and Methods

### Study species

The American Bullfrog *Lithobates catesbeianus* is a voracious and aggressive ranid frog that has been introduced beyond its native range (i.e. eastern North America) to at least thirty countries around the world (seven European countries among them) in the last two centuries [68–71]. Lowe et al. [72] catalogued *L. catesbeianus* as one of the world’s most pernicious invasive species, causing negative impacts in native amphibians in a variety ways [73–81]. Associated with the relatively large body size of *L. catesbeianus* (adult male snout-vent length [SVL] = 90–148 mm [82]), its advertisement call contains low spectral components (dominant frequency between 0.2–0.4 kHz and secondary frequency band between 1–2 kHz [83–85]), lower than those of the advertisement call of the most of Iberian native anurans (e.g. [86–88]), including Perez’s Frog *Pelophylax perezi* (adult male SVL = 40–85 mm; dominant frequency between 2.4–2.7 kHz [89,90]). The spectral characteristics of the call of *L. catesbeianus* are therefore likely to be advantageous over native species in terms of propagation efficiency. Moreover, *P. perezi* is a widespread Iberian ranid, the closest relative of *L. catesbeianus* in the Iberian Peninsula [91], and is considered to be one of the most successful species among Iberian anurans, with an almost continuous distribution throughout this region [92]. In the northernmost area of its range (i.e. south west France), *P. perezi* has occurred in sympatry with nonindigenous populations of *L. catesbeianus* for decades [69,92]. Both *L. catesbeianus* and *P. perezi* are aquatic-egg layers that congregate in water bodies where they form choruses during the breeding season [89,90,93–95]. Thus, *L. catesbeianus* and *P. perezi* are appropriate comparative models for assessing the competitive abilities in acoustic transmission between competing invasive and native species.

### Study sites

**Figure 1:**
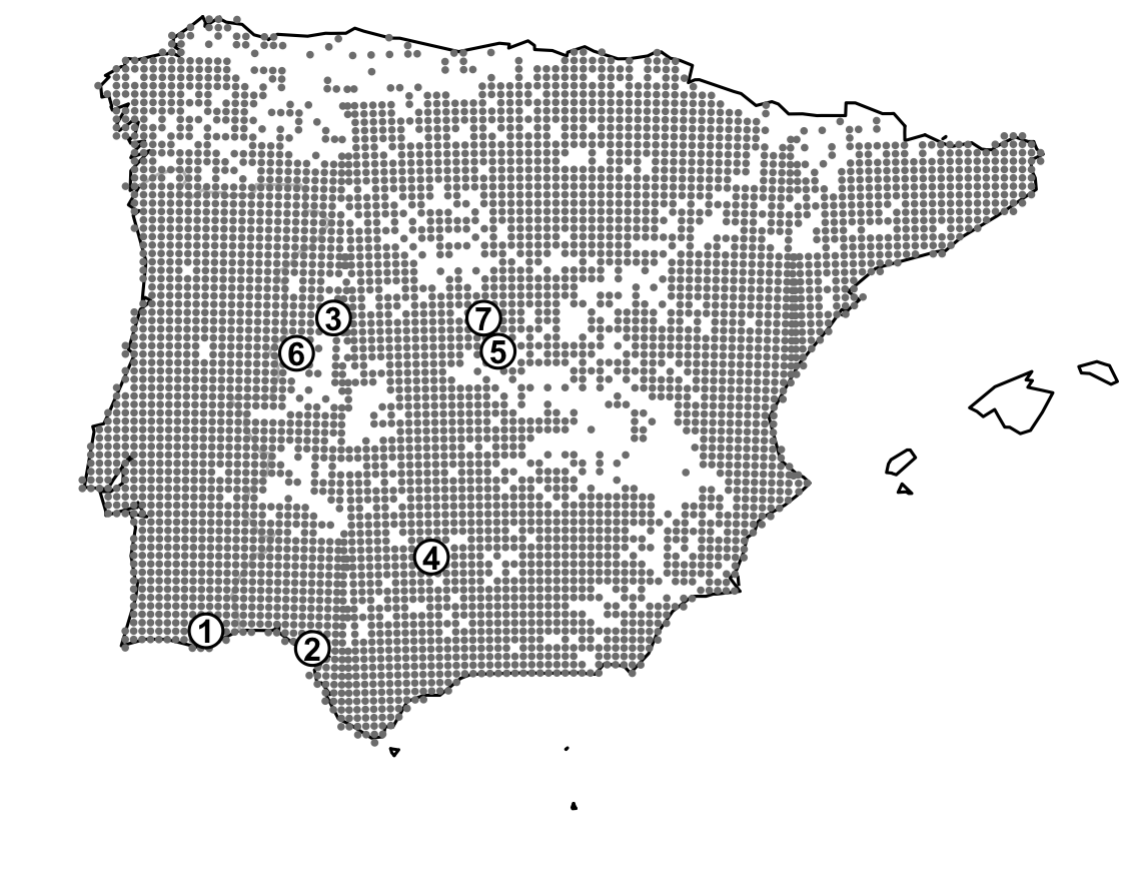
Distribution range of *Pelophylax perezi* and location of the study sites in the Iberian Peninsula. Grey circles correspond to the 10 x 10 km UTM squares (Universal Transverse Mercator coordinate system) with occurrence of *P. perezi* [136,137]. White circles correspond to the study sites: 1) Arimbo, 2) Doñana, 3) El Cabaco, 4) Las Jaras, 5) Navalcarnero, 6) Villasbuenas de Gata, and 7) Zarzalejo.

Sound propagation experiments were conducted in seven localities within the distribution of *Pelophylax perezi* in the Iberian Peninsula (Table 1, Figure 1). Study sites were selected to include different types of habitats where *P. perezi* breeds, such as rivers, creeks, marshes, and ponds. Measurements at three sites (El Cabaco, Las Jaras and Zarzalejo) were conducted in permanent ponds (0.1–0.9 ha) surrounded by grass pastures, bushes (*Rosa* spp.) and open Mediterranean forests (dominated by *Quercus pyrenaica*, *Pinus pinea*, and *Quercus ilex* and *Fraxinus angustifolia*, respectively). The substrate in El Cabaco was also partly rocky and bare. Two sites in Central Spain (Navalcarnero and Villasbuenas de Gata) included river shores having vegetation coverage of grass pastures and riparian forests (*Populus nigra* and *Fraxinus angustifolia*). The test site in Doñana was a temporary pool (1.6 ha) in the coastal salt marshes of the Doñana Biological Station. The water surface over which propagation measurements were made in Doñana had sedges (*Scirpus* spp.) and halophytic vegetation. Experiments in Arimbo were conducted in a creek with rocky shores and grass pastures. In two localities (Navalcarnero and Villasbuenas de Gata) *Lithobates catesbeianus* was introduced in the past from abandoned commercial farms, but established populations have not been confirmed [96–98]. 

**Table 1:** Environmental conditions in the study sites of propagation of advertisement calls of *Pelophylax perezi* and *Lithobates catesbeianus* in the Iberian Peninsula.

**Locality**	**Lat (N), Long (W)**	**Test date**	**Air temp. (°C)**	**Humidity (%)**	**Habitat**
Arimbo	37°10', 7°51'	30/03/09	6–11	90–96	Creek, rocky shore, grass pastures
Doñana	36°59', 6°26'	26/03/09	17–23	51–78	Coastal salt marshes, sedges
El Cabaco	40°32', 6°09'	03/06/09	16–19	52–87	Pond, bare soil, grass pastures
Las Jaras	37°58', 4°50'	01/04/09	14–20	47–70	Pond, bushes, open forests
Navalcarnero	40°10', 3°57'	11/06/09	15–23	65–94	River, grass pastures
Villasbuenas de Gata	40°09', 6°39'	01/06/09	26–30	35–77	River, riparian forests
Zarzalejo	40°32', 4°08'	04/06/09	12–23	44–78	Pond, bushes, grass pastures

Measurements were recorded between March and June of 2009, corresponding to the breeding season of the study species in this region, so that the study sites showed the same environmental conditions in which calling activity takes place. To avoid interference from calling individuals, the propagation experiments were conducted a few hours before sunset, prior to the peak of calling activity of the resident population. As air temperature and humidity have negligible effects on sound propagation at the frequencies and distances from the source that we tested [41,99,100], these measurements are assumed to apply also to night hours. During the experiments, the atmospheric conditions were stable and only slight gusts of wind occurred occasionally, during which measurements were temporally suspended. Air and surface water temperature and relative humidity were measured every 5 min with data loggers (HOBO Pendant 64K and HOBO Pro V-2, Onset Computer Corporation, Cape Cod, MA, USA) during the entire testing period. Background noise was also recorded for 45 s before and after the measurements with the microphone of a sound level meter (B&K 2238 Mediator, Brüel & Kjær, Nærum, Denmark) positioned on the propagation transects.

### Ethical statements

This study was carried out in public and protected areas. Permits to work and use of facilities in the protected area Doñana Biological Station were granted by ICTS Doñana (CSIC). Experiments did not include animal collection, capture, manipulation, or disturbance, endangered or protected species, and only involved the emission of recorded sounds. Therefore, this study was not submitted to approval for Institute of Animal Care and Use Committee (IACUC), and no specific permits were required according to national and local regulations. 

### Broadcast signals

Propagation efficiency of the study species were measured by pre-recorded signals played back through loudspeakers installed in natural environments, as used in previous studies (e.g. [32,33,40,41,53,101]). This experimental procedure was selected because it has shown lack of significant differences in attenuation average rates by comparing with those obtained from natural calls [102]. 

**Figure 2:**
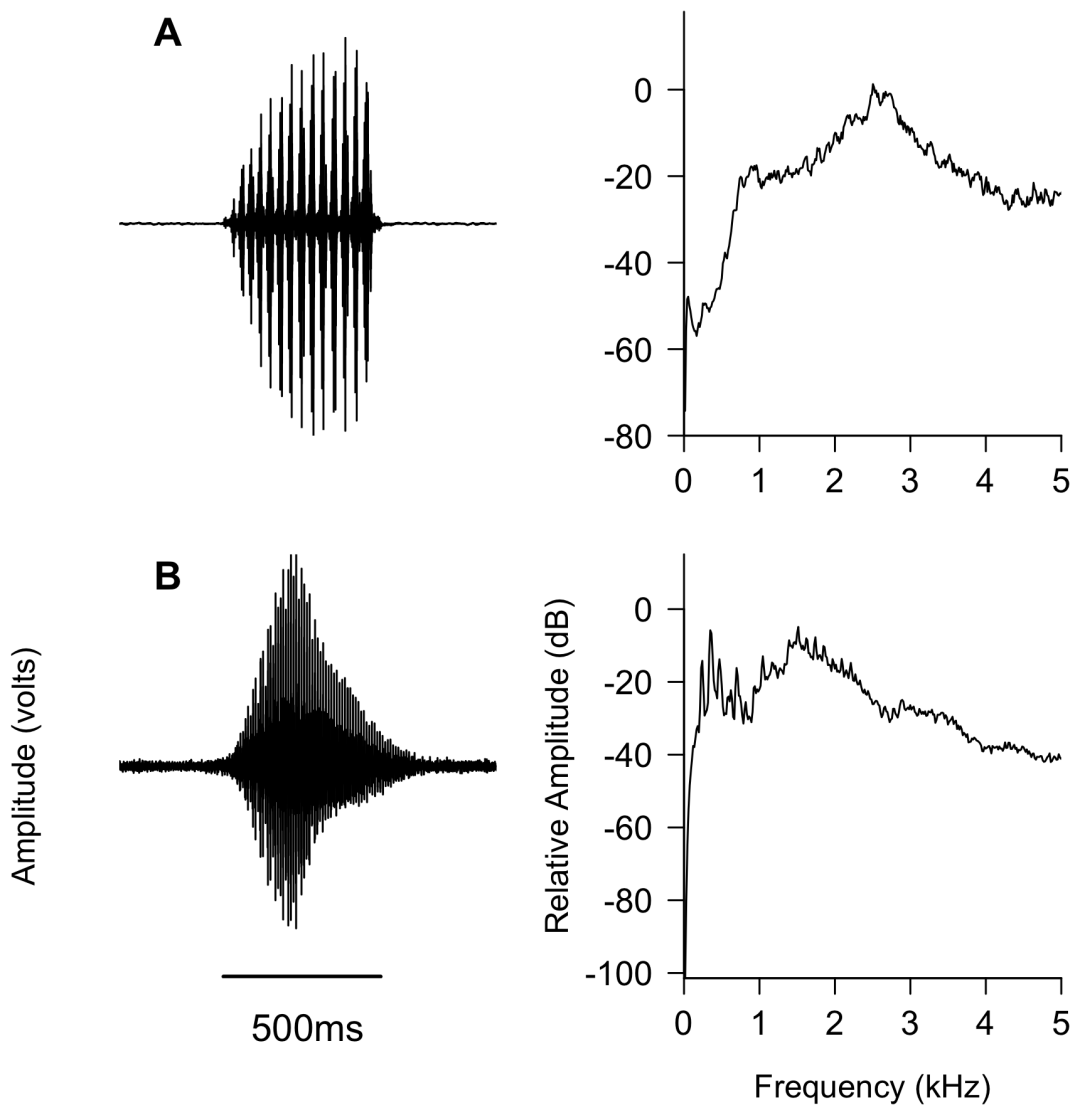
Oscillograms and power spectra of representative advertisement calls for sound propagation experiments: (A) *Pelophylax perezi* and (B) *Lithobates catesbeianus*. Air temperatures during recordings of these calls were 20 °C and 24 °C, respectively. Figures generated with Seewave software (3170 FFT size, 90% overlap, A-weighting; [105]).

A 3 min audio file (44.1 KHz and 16 bit) containing advertisement calls of 7 *Pelophylax perezi* and 6 *Lithobates catesbeianus*, 29 pure tones, and white noise was used for propagation experiments. The calls of *P. perezi* (Figure 2a) were recorded with a digital audio tape recorder (DA-P1 TASCAM, Montebello, CA, USA) and directional microphones (Sennheiser M66 and M80, Wedemark, Germany) between May and June of 2004 in El Casar (Guadalajara, Spain; N 40° 42’, W 3° 25’) at air temperatures of 16–20 °C. The calls of *L. catesbeianus* (Figure 2b) were recorded with a digital audio field recorder (Marantz PMD 660, Kanagawa, Japan) and directional microphones (Sennheiser MKH 70, Wedemark, Germany; and Telinga Pro 6, Uppsala, Sweden) in May of 2008 in the Mammoth Cave National Park (Kentucky, USA; N 37° 09’, W 86° 06’) at air temperatures of 24 °C. The vocalizations were recorded 2–10 m from the calling individuals. From each individual, 6 calls were included in the playback recording to account for intra-individual variation. All signals were previously filtered between 0 and 300 Hz and 100% peak normalized to standardize their relative amplitude with Raven 1.2 (Cornell University, Ithaca, NY, USA). For *P. perezi*, mean ± SD (minimum-maximum) of dominant frequency (Hz) and call duration (ms) were 2678.3 ± 279.5 (2153.3–3186.9) and 430 ± 183 (181–862), respectively. For *L. catesbeianus*, mean ± SD (minimum-maximum) of dominant frequency (Hz) and call duration (ms) were 1405.2 ± 552.6 (430.7–1981.1) and 641 ± 101 (453–862), respectively. These recordings are deposited in the collection of the Fonoteca Zoológica (Zoological Record Library of the National Museum of Natural Sciences, MNCN-CSIC, Madrid, Spain), collection codes 9086–9092 (*P. perezi*) and 8188–8189 (*L. catesbeianus*). In addition to the advertisement calls, 29 distinct pure tones of 0.5 s each and white noise of 3 s were also included in the playback recording in order to examine the transmission properties of the study sites for specific sound frequencies. The series of pure tones was composed of 23 tones between 200 and 2500 Hz, in steps of 100 Hz, and 6 tones between 2500 and 5000 Hz, in 500 Hz intervals. Signal generation and call editing was performed with Audacity 1.3.6 (SourceForge) and Peak Pro 5.2 (BIAS, Petaluma, CA, USA).

### Experimental procedures

The playback calls and pure tones were broadcast with a self-powered loudspeaker (Explorer Pro 7500, Anchor Audio, Carlsbad, CA, USA) connected to a laptop computer (Mac iBook G4, Apple Inc., Cupertino, CA, USA) and placed at positions typically occupied by calling males of *Pelophylax perezi* on the shores of water bodies at the study sites. The broadcast level was adjusted by setting a 1 kHz pure tone at 75 dB Peak SPL (dB re 20 μPa) at 0.5 m, measured with a sound level meter B&K 2238 Mediator (Brüel & Kjær, Nærum, Denmark). Distortion products measured for pure tones at 0.5 m from the loudspeaker were at least 30 dB below the amplitude of the tones generated. The frequency response of the loudspeaker was within ± 8 dB between 0.2 and 5 kHz (within ± 3 dB between 0.2 and 1.1 kHz and within ± 5 dB between 1.2 and 5 kHz). The unequal frequency response of the loudspeaker did not affect measurements, since attenuation was obtained by subtracting amplitudes of signals at a given distance from amplitudes of the same signal at 0.5 m from the loudspeaker (see below).

The broadcast signals were recorded with the microphone of a sound level meter (B&K 2238 Mediator, Brüel & Kjær, Nærum, Denmark) fitted with a 10-meter extension cable, a foam wind-shield and a digital audio field recorder (Marantz PMD-660, Kanagawa, Japan), and placed successively at distances of 0.5, 1, 2, 4, and 8 m from the loudspeaker in two type of substrates: water and soil. Measurements on the water substrate were conducted with the microphone supported on a tripod and positioned 5–10 cm above the water surface of the breeding habitats. Measurements on the soil substrate were conducted with the microphone placed 5–10 cm above the land of the surrounding area of the water bodies. The distances between the microphone and the loudspeaker were measured using a laser distance meter (Leica DISTO Classic5, Leica Geosystems AG, Heerbrugg, Switzerland). These distances were chosen to facilitate calculations of excess attenuation at distances doubling the preceding one and to allow comparisons with previous studies [38,41,102]. The recording level of the audio recorder was kept constant for all measurements, and at distant locations from the loudspeaker the sensitivity of the sound level meter was increased in the discrete steps provided by the instrument, in order to input detectable signals into the audio recorder. To calibrate the recordings, a 1-kHz pure tone at 94 dB SPL from a portable calibrator (B&K 4231, Brüel & Kjær, Nærum, Denmark) was recorded at the beginning and end of each transect. At the end of the playback, 3 s of white noise was emitted to check congruence with attenuation of specific frequency tones, and 1 min of background noise was recorded to estimate the signal-to-noise ratio.

### Signal analysis

Signal attenuation is a major effect of sound transmission that may interrupt effective communication if signals are attenuated below auditory thresholds of recipients. Thus, excess attenuation (i.e. attenuation in excess of that expected due to spherical spreading) of pure tones and playback calls was used as measure of sound propagation [31–33,38,40,41,102] and was calculated for distances of 1, 2, 4, and 8 m, relative to measurements at 0.5 m. First, sound amplitudes of the playback calls and pure tones recorded in propagation experiments were measured using Sound Ruler software [103]. Sound pressure levels (SPLs; dB re 20 μPa) of the recorded signals were determined relative to the value of the recorded calibration tone. Second, values predicted by spherical spreading were calculated with the equation: spherical transmission loss (dB) = 20 log [dB RMS SPL at far distance (m) / dB RMS SPL at 0.5 (m)]. Finally, these values were subtracted from the actual transmission loss (i.e. the differences between SPLs at 0.5 m from the loudspeaker and those measured at the corresponding farther distances). Positive and negative values of excess attenuation indicated that the sound attenuated at higher and lower rates, respectively, relative to the SPLs predicted by spherical transmission loss for each distance. In addition, maximum dB RMS and peak SPL of the background noise were calculated for 15 s periods of the noise recording. 

### Statistical analysis

To examine the physical properties of the habitats in which sound transmission tests were carried out, differences in excess attenuation of pure tones among localities, substrates (water and soil), distances (1, 2, 4, and 8m), and frequencies of the pure tones were statistically compared using four-way repeated measures ANOVA (*P* < 0.05). The *frequency* factor was pooled in five categories to reduce the number of groups, and was added as a between-subjects factor. The categories of frequency band were selected based on the spectral structure of the advertisement calls of both study species: F1) pure tones between 0.2 and 0.5 kHz that corresponds to the fundamental frequency of the calls of *Lithobates catesbeianus*; F2) pure tones between 0.6 and 1 kHz that encompass the frequency band between the fundamental and dominant frequencies of *L. catesbeianus*; F3) pure tones between 1.1 and 2 kHz that corresponds to the dominant frequency of *L. catesbeianus*; F4) pure tones between 2.1 and 3 kHz that include the frequencies of the advertisement calls of *Pelophylax perezi*; and F5) pure tones between 3.5 and 5 kHz, with frequencies above the previous ranges. Excess attenuation values of pure tones that were entered into the ANOVA test corresponded to single measurements at each distance, substrate and study site.

To analyse the differences in excess attenuation of the playback calls, a similar four-way ANOVA was computed with repeated measures on three factors (distance, substrate and locality) and one between-subjects factor (species). The *species* factor allows assessing the differences in sound propagation between the advertisement calls from the native and the invasive species. For the statistical analysis, the excess attenuation values obtained for the six calls of each species at a given distance, substrate and study site were converted to a linear scale (N/m^2^) to calculate averages and then reconverted to decibels. 

All interactions between factors were also considered in the analyses of both pure tones and playback calls. Normality and sphericity assumptions were assessed with Mauchly’s test and Shapiro-Wilk test. The excess attenuation values were log transformed to attain normality. When the sphericity assumption failed, Huynh-Feldt corrected *P*-values were reported. The differences among levels of factors were evaluated using multiple comparisons with Bonferroni correction. Average linear scale values were used in statistical analyses and average decibel values in figure design. Statistical analyses were conducted using SPSS 19.0 software (IBM Corporation, Armonk, NY, USA). Figures were composed with R [104,105].

## Results

### Attenuation of pure tones

**Figure 3:**
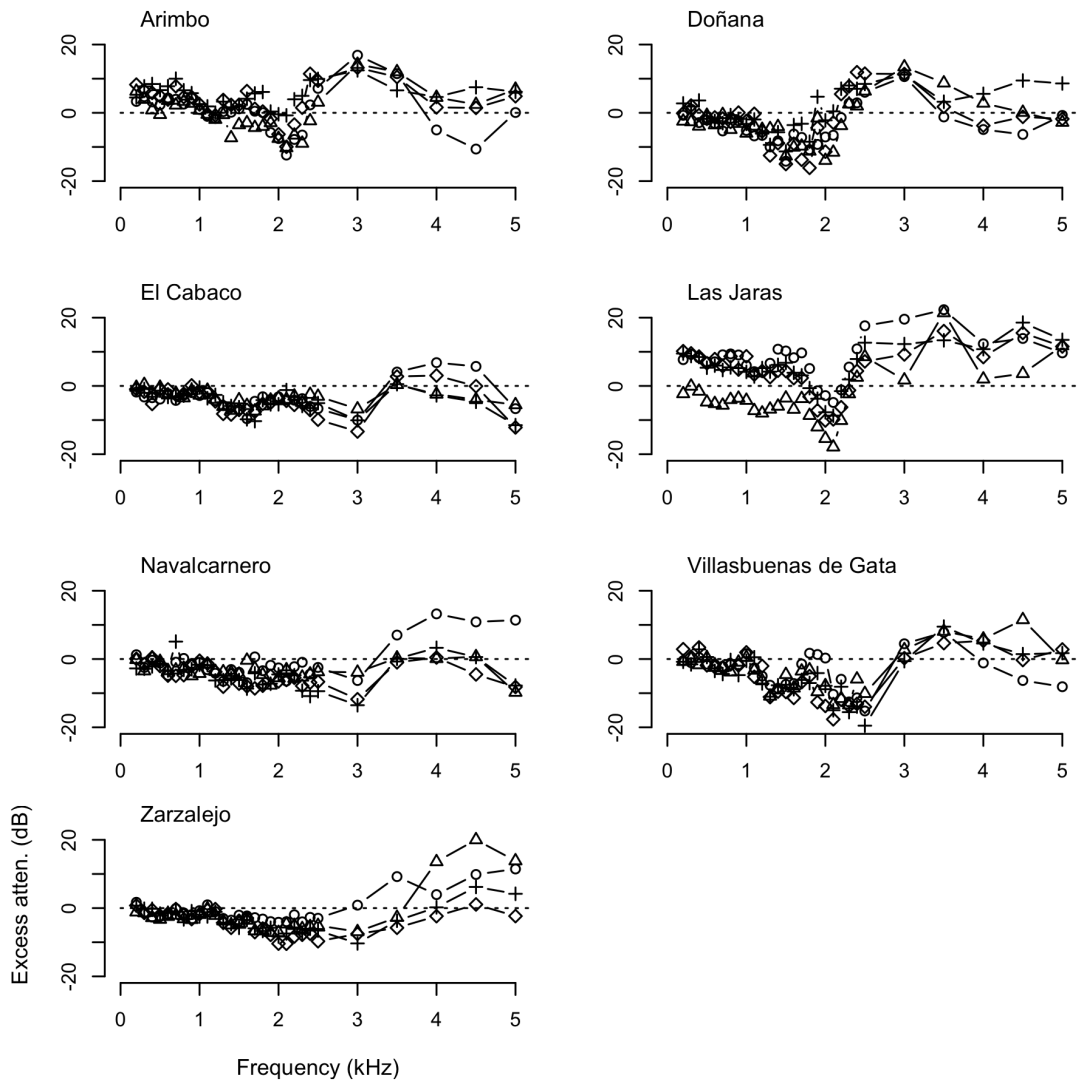
Excess attenuation (dB) of pure tones over water substrate in the study sites. Measurements recorded at 1 m (circles), 2 m (triangles), 4 m (crosses), and 8 m (diamonds) relative to SPLs (dB re 20 μPa) at 0.5 m from the loudspeaker.

**Figure 4:**
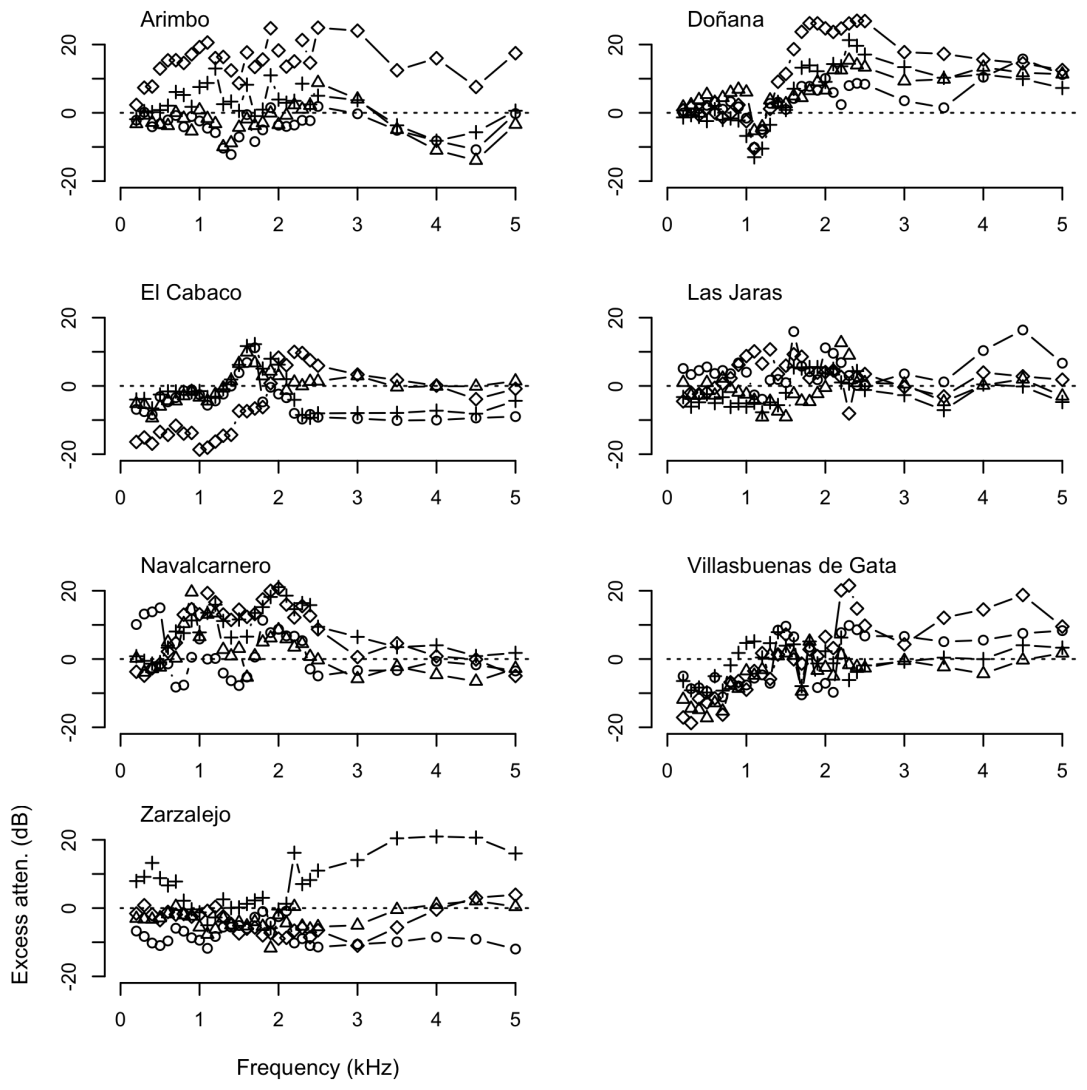
Excess attenuation (dB) of pure tones over soil substrate in the study sites. Measurements recorded at 1 m (circles), 2 m (triangles), 4 m (crosses), and 8 m (diamonds) relative to SPLs (dB re 20 μPa) at 0.5 m from the loudspeaker.

Measurements of propagation of pure tones showed that frequencies in the low range generally experienced lower attenuation rates, although the patterns of attenuation were markedly different across substrates and localities (Figures 3 and 4). Transmission of pure tones over water surface at frequencies below 2 kHz (F1–F3, within the spectral range of the call of *Lithobates catesbeianus*) resulted often in less attenuation that expected due to spherical spreading, with excess attenuation sometimes reaching values below -10 dB. However, excess attenuation showed a sharp increase at about 2–3 kHz (F4), corresponding to the dominant frequency of the advertisement call of *Pelophylax perezi* (Figure 3). In contrast with the water substrate, measurements of propagation of pure tones on the soil substrate were highly variable, with larger differences in attenuation among distances and study sites, and smaller differences among frequency categories, as shown in Figure 4. Mean, SD and range of excess attenuation of pure tones for each substrate and frequency category are summarized in Table 2.

**Table 2:** Excess attenuation (dB) of pure tones measured at four distances (1, 2, 4, and 8 m) on water and soil substrates of seven study sites in the Iberian Peninsula.

	**Frequency (kHz)**		**Excess attenuation (dB)**			
**Substrate**	**Categories**	**Range**	**Mean**	**SD**	**Min**	**Max**
Water	F1	0.2–0.5	1.76	5,61	-5.28	10.18
	F2	0.6–1.0	0.57	5,20	-5.97	9.99
	F3	1.1–2.0	-2.74	3,53	-16.12	10.76
	F4	2.1–3.0	1.49	1,68	-19.52	19.55
	F5	3.5–5.0	6.71	0,05	-12.16	22.33
Soil	F1	0.2–0.5	0.22	0,12	-18.75	15.00
	F2	0.6–1.0	2.70	1,03	-18.65	19.60
	F3	1.1–2.0	6.57	2,80	-18.09	26.22
	F4	2.1–3.0	9.39	2,77	-11.41	26.96
	F5	3.5–5.0	6.26	1,13	-13.95	20.97
Total	F1	0.2–0.5	1.02	2,43	-18.75	15.00
	F2	0.6–1.0	1.70	0,33	-18.65	19.60
	F3	1.1–2.0	3.11	3,82	-18.09	26.22
	F4	2.1–3.0	6.31	3,69	-19.52	26.96
	F5	3.5–5.0	6.49	0,58	-13.95	22.33

The frequency categories F1 and F3 correspond to the spectral range of the advertisement call of *Lithobates catesbeianus*, while F4 corresponds to that of the advertisement calls of *Pelophylax perezi*. The average excess attenuation data were not calculated directly from the averages of SPLs (dB re 20 μPa), but were obtained after transforming dB values to a linear scale and then converting back to dB.

The repeated measures ANOVA revealed that three of the factors considered (frequency, distance, and locality) showed significant effects on the attenuation rates of pure tones (Table 3). The frequency of the pure tones had a considerable influence on excess attenuation (*F*_4, 24_ = 17.70, *P* < 0.001). The lower frequency categories exhibited the lowest attenuation rates (mean = 1.02 dB for F1; mean = 1.70 dB for F2; mean = 3.11 dB for F3), and differed significantly in attenuation from the higher frequency categories (*P* < 0.05, in all cases; mean = 6.31 dB for F4; mean = 6.49 dB for F5). Transmission of pure tones was also affected by the distance (*F*_3, 72_ = 23.06, *P* < 0.001). Measurements of propagation at the largest distance (8 m) showed a significant increase in the rates of excess attenuation relative to shorter distances (*P* < 0.001, in all cases). Among the study sites, significant differences were found between El Cabaco, Villasbuenas de Gata, and Zarzalejo and the rest of localities (*P* < 0.01) and between El Cabaco and Zarzalejo (*P* = 0.014), the attenuation of pure tones being similar in the remaining of pairwise comparisons. Overall, the excess attenuation was similar between water and soil substrates (*F*_1, 24_ = 3.15, *P* = 0.089), but they largely differed in interaction with the rest of the variables (*P* < 0.001). Moreover, all interactions among factors in the model were also highly significant (Table 3).

**Table 3:** Four-way repeated measures ANOVA of the excess attenuation (dB) of pure tones in the study site of propagation of advertisement calls of *Pelophylax perezi* and *Lithobates catesbeianus* in the Iberian Peninsula.

**Factor**	***F***	**df**	***P***
Frequency	17.70	4, 24	< 0.001
Distance	23.06	3, 72	< 0.001
Substrate	3.15	1, 24	0.089
Locality	37.13	6, 144	< 0.001
Locality * Frequency	6.76	24, 144	< 0.001
Locality * Substrate	36.02	6, 144	< 0.001
Locality * Distance	32.14	18, 432	< 0.001
Substrate * Frequency	6.56	4, 24	0.001
Substrate * Distance	28.49	3, 72	< 0.001
Distance * Frequency	13.76	12, 72	< 0.001

Huynh-Feldt corrected *P*-values were reported when the sphericity assumption failed, as shown by Mauchly’s test.

### Attenuation of advertisement calls

**Figure 5:**
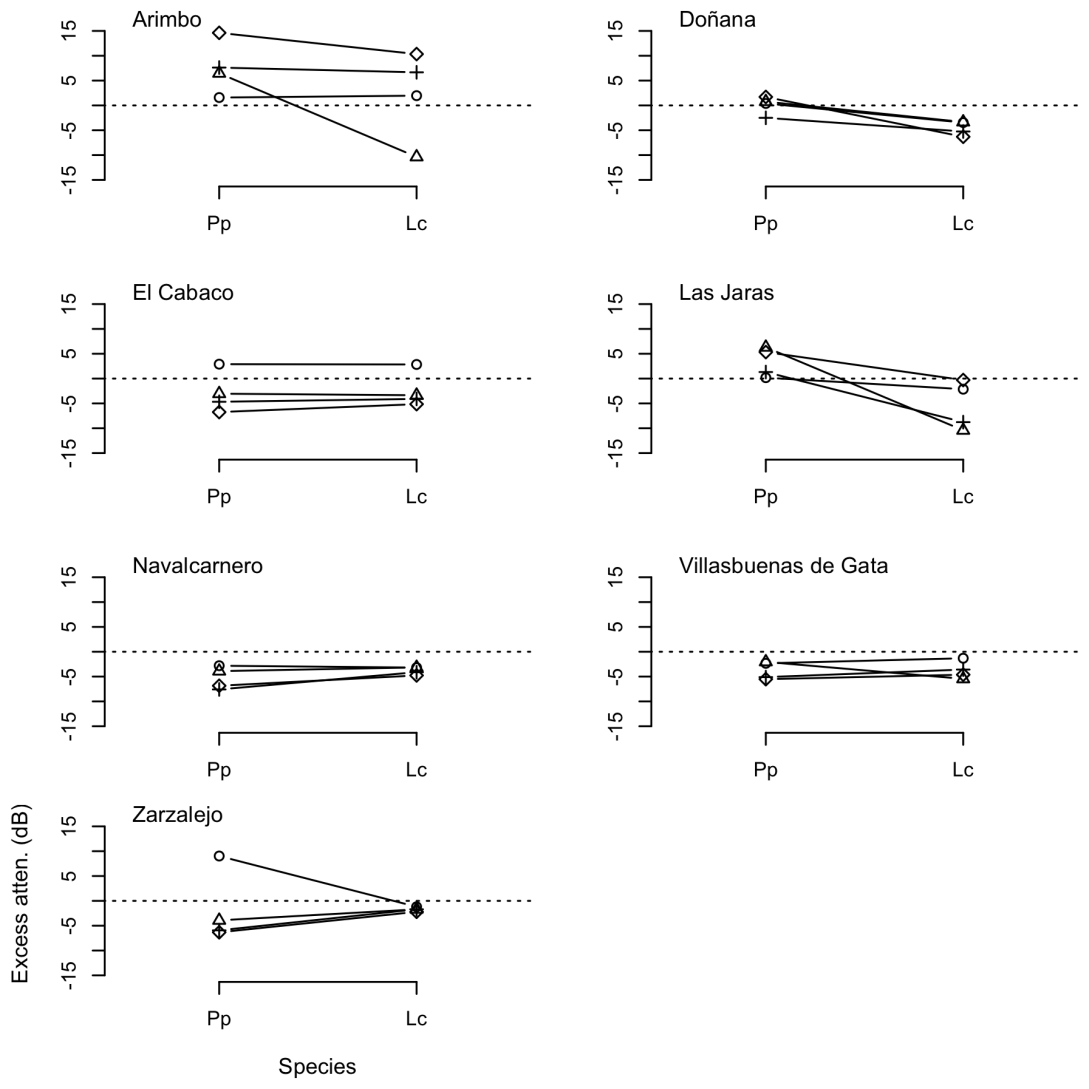
Excess attenuation (dB) of playback calls over water substrate in the study sites. Measurements recorded at 1 m (circles), 2 m (triangles), 4 m (crosses), and 8 m (diamonds) relative to SPLs (dB re 20 μPa) at 0.5 m from the loudspeaker. Each symbol represents the average for six calls of a species at a given distance. Abbreviations: Pp: *Pelophylax perezi*, Lc: *Lithobates catesbeianus*.

**Figure 6:**
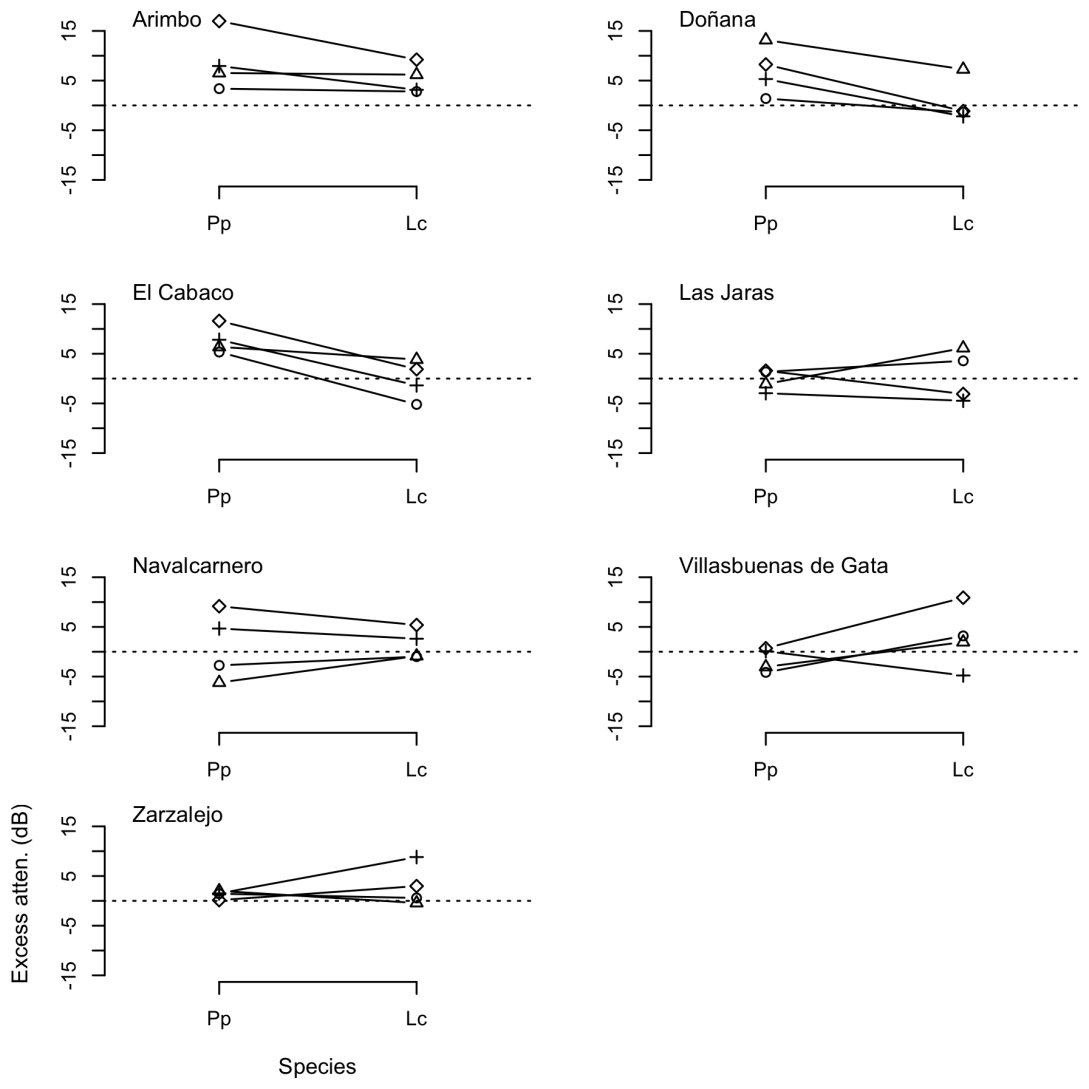
Excess attenuation (dB) of playback calls over soil substrate in the study sites. Measurements recorded at 1 m (circles), 2 m (triangles), 4 m (crosses), and 8 m (diamonds) relative to SPLs (dB re 20 μPa) at 0.5 m from the loudspeaker. Each symbol represents the average for six calls of a species at a given distance. Abbreviations: Pp: *Pelophylax perezi*, Lc: *Lithobates catesbeianus*.

Attenuation of playback calls followed patterns concordant with measurements of pure tones (Figures 5 and 6). Signals with lower spectral components, namely the advertisements calls of *Lithobates catesbeianus*, present lower overall attenuation rates, although patterns of propagation were largely variable depending on localities, substrates, and distances. Mean, SD and range of excess attenuation of advertisement calls of both species for each substrate and distance are summarized in Table 4.

**Table 4:** Excess attenuation (dB) of advertisement calls of *Pelophylax perezi* and *Lithobates catesbeianus* measured at four distances (1, 2, 4, and 8 m) on water and soil substrates of seven study sites in the Iberian Peninsula.

			**Excess attenuation (dB)**			
	**Substrate**	**Distance (m)**	**Mean**	**SD**	**Min**	**Max**
*P. perezi*	Water	1	2.17	4,74	-5.82	10.60
		2	1.20	3,66	-5.95	10.72
		4	-0.80	1,89	-9.52	9.30
		8	3.42	1,89	-8.82	18.13
		mean	1.63	6,83		
	Soil	1	1.37	8,71	-4.90	7.39
		2	4.75	2,08	-9.87	14.78
		4	4.25	6,34	-4.45	11.25
		8	8.96	2,43	-1.66	17.91
		mean	5.27	7,91		
*L. catesbeianus*	Water	1	-0.62	10,79	-4.11	3.15
		2	-4.82	9,43	-11.39	-1.27
		4	-1.64	3,10	-9.78	8.02
		8	0.28	0,72	-7.15	11.03
		mean	-1.50	7,76		
	Soil	1	0.82	10,10	-6.03	4.11
		2	3.96	8,15	-1.73	10.46
		4	1.53	4,24	-5.28	9.76
		8	5.02	4,97	-4.12	12.41
		mean	3.00	14,23		

The average excess attenuation data were not calculated from the averages of SPLs (dB re 20 μPa), but were obtained after transforming dB values to a linear scale and then converting back to dB.

**Figure 7:**
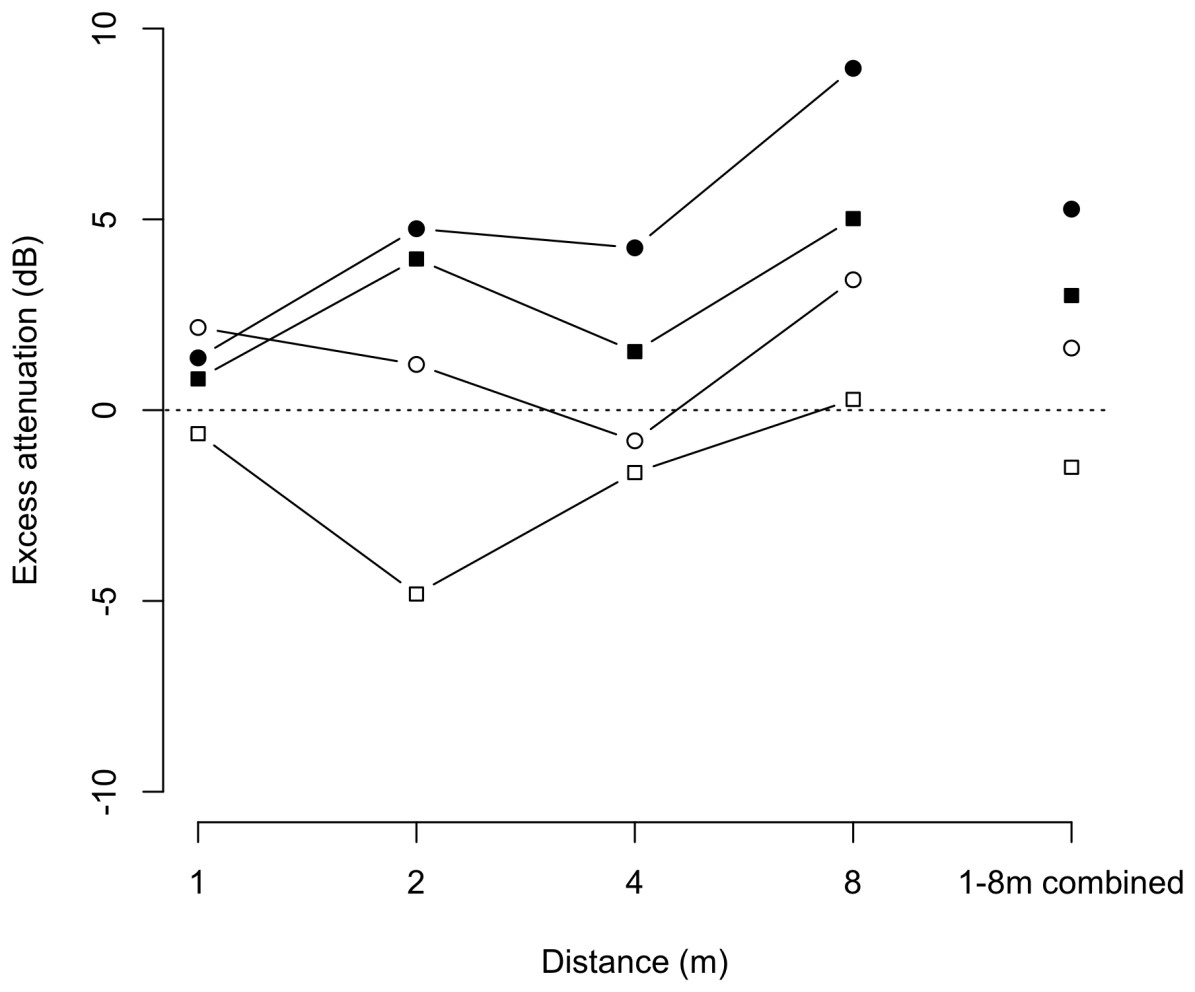
Averages of excess attenuation (dB) of playback calls for the seven study sites. Measurements for *Pelophylax perezi* (circles) and *Lithobates catesbeianus* (squares) emitted over water (open symbols) and over soil (filled symbols) substrates.

Excess attenuation of advertisement calls differed significantly across species, substrate, distance, and locality, as indicated by four-way repeated measures ANOVA (Table 5). The calls of *L. catesbeianus* experienced significantly lower attenuation rates than those of *Pelophylax perezi* (*F*_1, 11_ = 22.87, *P* = 0.001). Mean ± SD (minimum-maximum) of excess attenuation was 1.04 ± 2.6 dB (-11.39–12.41 dB) for *L. catesbeianus*, while 3.64 ± 2.5 dB (-9.87–18.13 dB) for *P. perezi*. As shown in Figure 7, these differences were found both for water and soil substrates (i.e. interaction between species and substrate: *F*_1, 11_ = 1.36, *P* = 0.267). Nevertheless, the interaction between species and site was found to be significant (*F*_6, 66_ = 34.17, *P* < 0.001), and the calls of *P. perezi* showed on average better propagation in riparian habitats, such as Villasbuenas de Gata and Navalcarnero, while the calls of *L. catesbeianus* suffered less attenuation in ponds or marshes, such as El Cabaco, Las Jaras, and Doñana (Figures 5 and 6).

**Table 5:** Four-way repeated measures ANOVA of the excess attenuation (dB) of playback calls in the study site of propagation of advertisement calls of *Pelophylax perezi* and *Lithobates catesbeianus* in the Iberian Peninsula.

**Factor**	***F***	**df**	***P***
Species	22.87	1, 11	0.001
Substrate	227.47	1, 11	< 0.001
Distance	202.21	3, 33	< 0.001
Locality	92.98	6, 66	< 0.001
Locality * Species	34.17	6, 66	< 0.001
Locality * Substrate	10.82	6, 66	0.001
Locality * Distance	142.49	18, 198	< 0.001
Substrate * Species	1.36	1, 11	0.267
Substrate * Distance	373.43	3, 33	< 0.001
Distance * Species	7.97	3, 33	< 0.001

Huynh-Feldt corrected *P*-values were reported when the sphericity assumption failed, as shown by Mauchly’s test.

The ANOVA revealed that type of substrate was the factor with the greatest effect (*F*_1, 11_ = 227.47, *P* < 0.001), showing water surface as a substrate with remarkably better proprieties for sound transmission than soil substrate in both species (Figure 7). Excess attenuation of playback calls was also highly affected by distance (*F*_3, 33_ = 202.21, *P* < 0.001). Differences in attenuation were found among all positions within the propagation transect (*P* < 0.01, in all cases), with larger excess attenuation at the farthest distance between sound source and recipient (8 m; Figure 7). Significant interactions were found among all the factors considered, except the latter mentioned interaction between species and substrate. The relationship between excess attenuation and playback calls of both species varied among distances and localities (Table 5).

## Discussion

Despite being broadcast in nonindigenous habitats, the advertisement calls of the invasive species were, overall, transmitted more efficiently than those of the native species, as shown by sound propagation experiments in different environments. Lower attenuation rates were found for acoustic signals of *Lithobates catesbeianus* in both aquatic and terrestrial substrates, and in most of the study sites. The less efficient propagation of the signals of the native species suggests an absence of optimal relationships between native environments and propagation of acoustic signals, in contrast to the acoustic adaptation hypothesis [31,35], proposed from evidence in other vertebrates. Our results are in general agreement with an increasing number of studies examining this prediction in other anurans from temperate and tropical habitats that have not found relationships between habitat structures and call characteristics [37–39,41,61,106–108]. In anurans, this apparent lack of optimization is likely related to the restricted distances over which anurans communicate as compared to birds or mammals [27]. Thus, our findings imply that adaptive mechanisms optimizing sound transmission in native habitats may play a less significant role than other selective forces or biological constraints in evolutionary design of acoustic signal in anurans. This suggestion has previously been proposed for tropical anuran species [48,61], and is also concordant with findings in some bird species [47,67,109]. 

The different call transmission efficiency between the invasive and native species may be due to the lower frequency contents of the spectra of the advertisement calls of the *L. catesbeianus* (with spectral peaks at about 0.5 and 1.8 kHz) relative to those of the *Pelophylax perezi* (with peaks at about 1 and 3 kHz). As revealed by the analysis of propagation of pure tones, the observed patterns of call transmission were consistent with properties of the different study sites for sound propagation over different frequency ranges. It has been well established that particular spectral and temporal features, may increase attenuation and degradation process of acoustic signals through environment, regardless of habitat structure [30,32,33,35–37,39,41,110]. In general, higher frequency sounds experience more attenuation over distance than lower frequency sounds, and amplitude-modulated signals restrict sound transmission relative to tonal signals (e.g. [32,34]).

Sound propagation experiments of the present study rather than being consistent with the acoustic adaptation hypothesis support the morphological constraints hypothesis [47,48]. This hypothesis proposes that morphological features, such as body size, mass of vocal cords or size of other sound production structures, which evolve from multiple concomitant selective pressures, exert a more substantial influence on call spectral parameters, and hence on call transmission efficiency, than mechanisms of signal adaptation optimizing transmission efficiency in native habitats. Body size is a major factor influencing spectral properties of the advertisement calls, as it affects the size of sound production mechanisms, so that spectral properties of signals are usually a function of body size both within and among species using similar sound production mechanisms [27,48,61,66,67]. As *L. catesbeianus* is around twice the averaged size of *P. perezi* [82,89,90], the spectral differences between acoustic signals of the two species are presumably due to differences in body size. As such, the smaller body size of the native species relative to invasive species imposes constraints to the propagation of the advertisement calls even within its indigenous range.

The lack of a strict correspondence between signal quality and environment is likely to render native sound-communicating vertebrates particularly vulnerable to intrusion generated by invasive species producing low frequency signals of high amplitude, such as *L. catesbeianus*. The efficient signal propagation of its advertisement calls is likely to confer a competitive advantage to this invasive species in acoustic communication and reproduction, favouring processes of establishing and spreading outside their native range. Acoustic communication plays a crucial role in species recognition and mate attraction and selection in anurans and other acoustic communicating animals [25–28], and hence it is expected that a better call transmission efficiency results in an increase of the range of effective communication between conspecifics and of the probability of reproductive success. However, to confirm this prediction it would be necessary to assess the remaining features of the communication system of the study species, such as hearing thresholds, sound pressure levels of the advertisement calls, or sender-receiver distances within breeding choruses, given that they might counteract or increase the specific constraints in signal propagation. This information is available for *L. catesbeianus*, and indicates that this species is able to communicate over long distances of about 60 m (e.g. [111–115]). However, this information is not available for most of temperate anurans, including *P. perezi*, which prevents comparative analyses. Thus, future studies are needed to confirm that the properties of the advertisement call of *L. catesbeianus* may contribute to the invasion process. 

Nonindigenous populations of *L. catesbeianus* cause negative impacts in native amphibians due to direct predation [74–77], resource competition [73,76,80], and pathogen carriage, such as *Batrachochytrium dendrobatidis* [78,79,81]. Although there is growing evidence that the noise interference generated by abiotic sources [116–120], biotic sources [121–125], and anthropogenic sources [126–129] have diverse consequences for vocal activity of vertebrates, few studies have examined the possible consequences of acoustic competition of nonindigenous species. Such competition could be significant, especially if these species exhibit calls with (1) high sound amplitude, (2) frequency spectra overlapping native calls, and (3) low attenuation in their transmission through the environment. The advertisement calls of *L. catesbeianus* seem to combine these three characteristics. The first evidence suggesting an effect of the vocalizations of introduced *L. catesbeianus* on acoustic communication of native populations has recently been reported for the neotropical tree frog *Hypsiboas albomarginatus*, which increases call frequency in response to invasion of its acoustic niche by calls of *L. catesbeianus* [42]. Because of the female preference for lower frequency signals recorded in several anurans (e.g. [27,28,130–134]), a shift to higher frequencies could have a negative effect on the reproduction of native anurans. Moreover, interference also affects parameters of vocal activity other than frequency, such as amplitude, call duration, and rate of emission of vocalizations in different vertebrates (e.g. [135]), effects that may be experienced by native sound–communicating communities confronting foreign acoustic intrusions. Future studies should provide more extensive assessments of effects of such potential exposures. 
